# Associations Between Affective States and Sexual and Health Status Among Men Who Have Sex With Men in China: Exploratory Study Using Social Media Data

**DOI:** 10.2196/13201

**Published:** 2020-01-31

**Authors:** Zhi-Wei Zheng, Qing-Ling Yang, Zhong-Qi Liu, Jia-Ling Qiu, Jing Gu, Yuan-Tao Hao, Chao Song, Zhong-Wei Jia, Chun Hao

**Affiliations:** 1 Department of Medical Statistics School of Public Health Sun Yat-sen University Guangzhou China; 2 Health Information Research Center, Guangdong Key Laboratory of Medicine School of Public Health Sun Yat-sen University Guangzhou China; 3 Sun Yat-sen Global Health Institute Institute of State Governance Sun Yat-sen University Guangzhou China; 4 School of Computer Science and Engineering University of Electronic Science and Technology of China Chengdu China; 5 National Institute on Drug Dependence Peking University Beijing China

**Keywords:** affect, men who have sex with men, sexual behaviors, health status, social media

## Abstract

**Background:**

Affective states, including sentiment and emotion, are critical determinants of health. However, few studies among men who have sex with men (MSM) have examined sentiment and emotion specifically using real-time social media technologies. Moreover, the explorations on their associations with sexual and health status among MSM are limited.

**Objective:**

This study aimed to understand and examine the associations of affective states with sexual behaviors and health status among MSM using public data from the Blued (Blued International Inc) app.

**Methods:**

A total of 843,745 public postings of 377,610 MSM users located in Guangdong were saved from the Blued app by automatic screen capture. Positive affect, negative affect, sexual behaviors, and health status were measured using the Simplified Chinese Linguistic Inquiry and Word Count. Emotions, including joy, sadness, anger, fear, and disgust, were measured using the Weibo Basic Mood Lexicon. A positive sentiment score and a positive emotion score were also calculated. Univariate and multivariate linear regression models on the basis of a permutation test were used to assess the associations of affective states with sexual behaviors and health status.

**Results:**

A total of 5871 active MSM users and their 477,374 postings were finally selected. Both positive affect and positive emotions (eg, joy) peaked between 7 AM and 9 AM. Negative affect and negative emotions (eg, sadness and disgust) peaked between 2 AM and 4 AM. During that time, 25.1% (97/387) of negative postings were related to health and 13.4% (52/387) of negative postings were related to seeking social support. A multivariate analysis showed that the MSM who were more likely to post sexual behaviors were more likely to express positive affect (beta=0.3107; *P*<.001) and positive emotions (joy: beta=0.027; *P*<.001), as well as negative emotions (sadness: beta=0.0443; *P*<.001 and disgust: beta=0.0256; *P*<.001). They also had a higher positive sentiment score (beta=0.2947; *P*<.001) and a higher positive emotion score (beta=0.1612; *P*<.001). The MSM who were more likely to post their health status were more likely to express negative affect (beta=0.8088; *P*<.001) and negative emotions, including sadness (beta=0.0705; *P*<.001), anger (beta=0.0058; *P*<.001), fear (beta=0.0052; *P*<.001), and disgust (beta=0.3065; *P*<.001), and less likely to express positive affect (beta=−0.0224; *P*=.02). In addition, they had a lower positive sentiment score (beta=−0.8306; *P*<.001) and a lower positive emotion score (beta=−0.3743; *P*<.001).

**Conclusions:**

The MSM social media community mainly expressed their positive affect in the early morning and negative affect after midnight. Positive affective states were associated with being sexually active, whereas negative affective states were associated with health problems, mostly about mental health. Our finding suggests the potential to deliver different health-related intervention strategies (eg, psychological counseling and safe sex promotion) on a social media app according to the affective states of MSM in real time.

## Introduction

### Background

Affective states, including emotion and sentiment, can be defined as positive or negative evaluations of objects, behaviors, or thoughts [1]. Affective states are critical determinants of health [2,3]. An emotion is a cognition that arouses one or more specific forms of a certain generic type of body reaction [4]. There are 6 basic emotions: joy, sadness, anger, fear, disgust, and surprise. For example, to fear something, such as a snake, is to be cognizing something fearful. The sentiment is a conscious state that develops over time from emotions [5] and is more stable and more permanent than emotions [6]. Sentiment is highly socialized. Suppose a certain object with a complex nature and structure has been repeatedly perceived or thought of by a person in many different contexts on various occasions, these various cognitions of the object will produce a complex dispositional idea of the object, finally leading to strong emotions being felt toward it on many occasions. Anything that excites the dispositional idea of the object will tend to excite all strong emotional dispositions. Summing up all these emotions forms a sentiment about this object. For example, an elderly person feels pride when he or she remembers his or her childhood days. The sentiment includes positive affect (eg, love and honor) and negative affect (eg, hurt and annoyance) [7]. Negative affect and negative emotions are common components of depression, whereas positive affect can facilitate positive health behaviors across the population [8,9]. Therefore, affective states are important components of mental health status to indicate or predict health-related behaviors.

In recent years, with the development of social media apps, an increasing number of studies have suggested that significant links exist between the indicators of emotional well-being or affective states and users’ behaviors based on an analysis of user-generated textual data. Golder et al [10] pioneered in using real-time user-generated textual data from Twitter and analyzing the daily changes of positive affect and negative affect, which was the flagship study involving social media data for analyzing emotion-related outcomes. Several following studies have shown the feasibility of using social media data to investigate various health-related issues in the general population, including sleep complaints, depression, anxiety, suicide, and HIV [11-14]. However, similar studies using social media data are rare for the HIV risk population, men who have sex with men (MSM). There was a study that used Grindr, which is a geosocial networking app for MSM in the United States [15]. However, the study used Grindr as the recruitment tool to recruit the MSM population and then conducted a Web-based survey via Grindr but did not derive results using the user-generated data in Grindr [15]. Therefore, there is a lack of studies assessing and monitoring the affective states of MSM by analyzing user-generated data on social media apps.

Blued, like Twitter and Facebook, is a social media site with an app, originating in China, for gay, bisexual men, transgender women, and MSM to communicate with each other to find sexual partners and share information [16,17]. In 2016, Blued reported that it had 27 million users, making it the most widely used gay app in China. There are indications that accessing Blued has become a daily activity for many MSM users: 20% spend at least two hours per day on Blued and 10% post over 200 messages per day, including postings and comments [18]. In addition, owing to its anonymity, MSM can talk freely on Blued about private issues, such as sexual behaviors, health, and HIV status [7,19]. These indicate the potential of using Blued as a valuable data source to assess and monitor affective states among MSM [11,12,20-22].

Using Blued to find male sex partners is related to sexual health and sexually transmitted diseases. There were some questionnaire-based studies on the associations between affective states and sexual behaviors among the general population. It has been found that positive affect and positive emotions (eg, joy) may increase sexual desire that can facilitate sexual behaviors [23], whereas one study has indicated that positive affect does not always translate into behaviors [24]. Similarly, negative affect and negative emotions (eg, sadness or anger) can facilitate or inhibit sexual behaviors [25,26]. For example, anxiety has been found to facilitate genital responses [26], whereas another study suggested that individuals who experienced negative emotions practiced fewer sexual behaviors than those who had positive emotions [25,26]. In the studies of MSM, those with depression or higher negative affect are more likely to practice condomless anal intercourse or have multiple sexual partners [27-29]. Researchers have revealed that affective states may influence the health status among MSM. For example, a depression symptom is closely related to HIV treatment nonadherence, and positive affects predict linkage to HIV care and antiretroviral therapy persistence [30,31]. However, these MSM studies were questionnaire based and with some opposite results compared with the general population.

Furthermore, mental health greatly depends on affective states [32]. It is found that individuals’ inner emotions have been shown to manifest in their choice of words in writing [33,34]. Moreover, studies showed that emotional words used on social media were associated with depression measured by psychological scales [35,36]. The mental health status measured by the questionnaire-based psychological scale needs to be regularly updated to stand the test of time. However, psychological support for depression and anxiety should be timely because of the mental disorder with rapid and unexpected changes. Evidence in mental health research suggests that depression and anxiety are highly prevalent among MSM, for whom depression rates were 40% and 37.6% in the United States and China, respectively [37-39]. Although there are some barriers in using textual data (occurrence of *good* preceded by *not*, recognition of emoticons, etc), it is still a cost-effective way to examine affective states and, therefore, design-specific interventions on psychological support based on social media data [10,40].

### Objectives

Therefore, this study was designed to explore affective states, including sentiment and emotions, and their associations with sexual behaviors and health status among Chinese MSM by analyzing user-generated data in Blued. Specifically, this study aimed to understand the following: (1) the description of affective states by displaying the diurnal rhythms of sentiment and emotions and (2) the associations of affective states with sexual behaviors and health status.

## Methods

### Data Collection

Guangdong is China’s most developed province, and it emerged as the earliest province with MSM using the internet in China. Internet-based dating started in Guangdong from the website, GZTZ.ORG, run by the MSM community named as Lingnan, since 1998 [41]. Therefore, the MSM in Guangdong have the longest history of internet-based data and are the largest number of Blued users [18] among all provincial-level divisions in China. Meanwhile, the MSM in China aggregate in the most developed areas [42]. Megacities provide MSM a friendly environment and plenty of opportunities to make friends with each other. Out of 4 megacities in China, 2 megacities, Guangzhou and Shenzhen (GDP per capita is similar to southern European countries), are in the Guangdong province, with a similar Cantonese culture. On the basis of these reasons, we chose the Guangdong province as the target area for our exploratory study. Therefore, from July 2013 to July 2016, 843,745 public postings (N) of 377,610 MSM users (U), including their public background information and postings on Blued, located in Guangdong, were culled from the Blued app through our application programming interface (API). All the data we culled were public information available on Blued, including public background information and postings. The public information data are anonymous. The related participants’ identified information has been strictly protected by Blued. In the Blued user agreement, the registered users have agreed that the content posted by users in this app may be copied, reposted, or used by third parties. This study itself does not involve any physical, social, or legal risks to the participants.

To conveniently control various variables involved in our data collection method such as the time interval between 2 simulated clicks, we packaged the three steps of our data collection method into an API. We designed a simulation-based data collection method with three steps: (1) We utilized the android debug bridge (ADB) to simulate the operation of a user using a social network app. Specifically, the ADB can drive a mobile phone to perform a series of actions on the user interface, such as swiping, clicking, or dragging. After these simulated operations, the data (eg, username and profile image) we wanted to collect were displayed on the screen. (2) We used automatic screen capture technology and optical character recognition (OCR) to extract the data displayed on the phone screen. For image data such as profile images and photos, we drew the display zone of each kind of image data according to the user interface of Blued. On the basis of the display zone, we captured the screenshot to obtain the image data we wanted. As for textual data such as username and age, we utilized an open-source OCR tool to extract the text displayed in the screenshot. It is necessary to note that our target texts were of printable style and always displayed in a fixed position in the screenshot. Therefore, the OCR in our method could get high accuracy. (3) We cleaned the data and stored them in a secure device. Owing to the sensitive nature of this work, we took careful steps to protect user privacy and ensure the ethics of the research. All users’ data were deidentified in our study. Specifically, we anonymized each user’s identity with a random user ID. All data were stored in a secure private server without public access. Only members of our research team had access to log in to this server. Data were transferred using encrypted Secure Sockets Layer connections to ensure that they cannot be intercepted by third parties.

**Figure 1:**
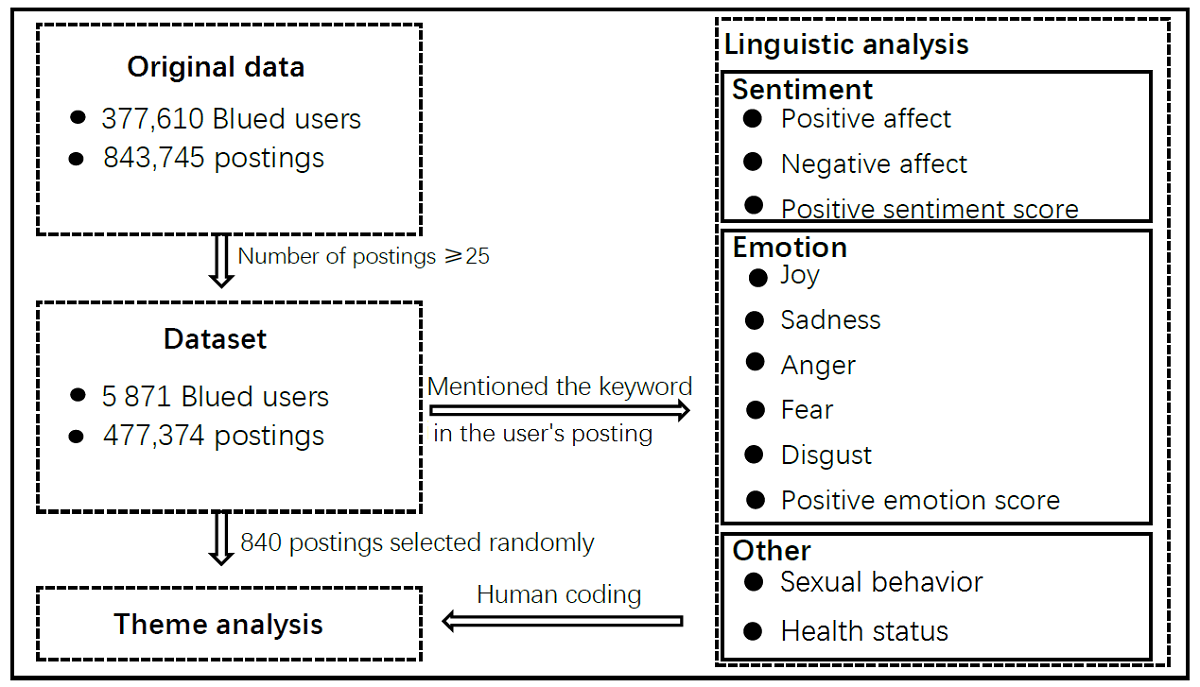
Flow chart of the data collection.

We grouped these data into five databases: user profile database, postings database, comments database, followers database, and followees database. The number of postings is one of the indicators to determine whether a user is an active user. Therefore, users with 25 postings or more were included in the data analysis [40]. Consequently, our sample for this study contained 5871 active users (U), including gay, bisexual men, and transgender women, and 447,374 postings (N; see Figure 1).

### Measures

#### Demographic and Social Network Characteristics

Demographic characteristics, including age, educational level, geolocation, hometown, height, weight, and sex role, were obtained from the user profile database. Social network characteristics, including the number of followers, followees, and chat groups, were also obtained from the user profile database. Age was categorized into *≤25 years old* and *>25 years old* based on the social and developmental psychology literature [11,43]. Educational level was classified as *high school or below*, *above high school*, and *unknown*. Geolocation was categorized into Guangzhou, Shenzhen, Dongguan, and other cities in the Guangdong province based on the distribution of the geolocation of the MSM. Hometown was classified as Guangdong, non-Guangdong, and unknown. Height and weight were transformed into BMI, which was associated with depression as well as sexual activity in the MSM community [44]. Blued users are more likely to search for partners who are in good shape [45]; therefore, BMI is an important indicator for their fitness. BMI was categorized into underweight (BMI<18.5 kg/m^2^), normal (18.5 kg/m^2^≤BMI<25 kg/m^2^), overweight (25 kg/m^2^≤BMI<30 kg/m^2^), and obese (BMI≥30) on the basis of the guidelines from the National Institutes of Health [44]. In addition, research suggested that those reporting high levels of depressive symptoms were more likely to report both receptive and insertive unprotected anal intercourse [46]. Therefore, sex role was classified into insertive, receptive, and versatile sexual roles and unknown [46]. A *chat group* was defined as an online forum that enables users to conduct instant message-based private conversations with other users. The number of followers, followees, and chat groups, which reflects the social network size or social capital, is associated with depression [47,48]. Owing to the skewed data, the number of followers, followees, and chat groups was log transformed for analysis [49].

#### Affective States: Sentiment and Emotion

##### Overview

To measure affective states, we first calculated the probability of words related to affect across all postings for each user in a given hour (P(u,h)). Then, we calculated each user’s baseline probability of affect (averaging P(u,h)) across all hours. To measure diurnal mood rhythms, we calculated the grand mean of affect across all users and the relative probability of affect per user. Finally, we calculated the general level of the relative probability of affect across users who were active during hour h. Owing to 7 dependent variables (positive affect, negative affect, and 5 emotions) and many independent variables, there was a possibility of difficulty in exploring the true relationships between them. Thus, to ensure that emotion and sentiment were expressed in one single measure by each posting, the positive emotion score and positive sentiment score were widely applied in different studies [7,50-52]. The calculation of the positive sentiment score and positive emotion score has been described in Step 1. A positive emotion score is different from a positive sentiment score. The true difference is about the concepts of sentiment and emotion. Emotions are preconscious social expressions of feelings and affect [53]. Sentiments are partly social constructs of emotions that develop over time and are enduring [53]. In other words, sentiments have been found to be held for a longer period and are more stable and dispositional than emotions [53]. Therefore, the word list of sentiment is different from the word list of emotion. For example, the word *honor* is a positive affect–related word but is not a joy-related word (Table 1).

**Table 1:** Summary of the Simplified Chinese Linguistic Inquiry and Word Count and Weibo Basic Mood Lexicon dimensions used for this study and example vocabulary. Example vocabulary in the original Chinese can be found in Multimedia Appendix 1A.

Dimensions		Dictionary	Number of words	Example vocabulary
**Sentiment**				
	Positive affect	SC-LIWC^a^	483	Honor, sweet, happy
	Negative affect	SC-LIWC	812	Hurt, agony, nasty
**Emotion**				
	Joy	Weibo-5BML^b^	306	Love, excited, high
	Sadness	Weibo-5BML	205	Anxious, alone, tear
	Anger	Weibo-5BML	93	Enemy, abuse, roar
	Fear	Weibo-5BML	72	Sit on pins and needles, panic, hell
	Disgust	Weibo-5BML	142	Wordy, speechless, ridicule
**Other**				
	Sexual-related words	SC-LIWC	117	Sex, condom, kiss
	Health-related words	SC-LIWC	375	Infection, insomnia, exercise

^a^SC-LIWC: Simplified Chinese Linguistic Inquiry and Word Count.

^b^Weibo-5BML: Weibo Basic Mood Lexicon.

##### Step 1

The probability of sentiment and emotions of postings was measured using the Simplified Chinese Linguistic Inquiry and Word Count (SC-LIWC) [54] and Weibo Basic Mood Lexicon (Weibo-5BML) [55]. Owing to the coverage of the word list, using the Chinese version of LIWC (SC-LIWC) was insufficient. Positive affect–, negative affect–, anger-, and sadness-related words are listed in the SC-LIWC. However, joy-, fear-, and disgust-related words are not included in the SC-LIWC. Fortunately, Weibo-5BML, which includes joy, sadness, anger, fear, and disgust, has been developed and can be used as a supplementary tool of LIWC. Therefore, we used SC-LIWC for affect and Weibo-5BML for emotions. A summary of SC-LIWC and Weibo-5BML dimensions’ example words for this study is presented in Table 1. This kind of measurement that uses different dictionaries in the same textual data has been applied in Hong’s study as well [56]. The validity of LIWC’s performance for sentiment has been shown in some studies [54,57-59]. In addition, the validity of Weibo-5BML’s performance for emotion has been shown in other studies [55,60,61]. Specifically, Weibo-5BML was validated by comparing the mood time series with fluctuations recorded and labeled by the vital social events and traditional festivals in China [55,60,61]. *Positive affect* and *negative affect* dimensions from SC-LIWC were selected to measure perceived positive and negative affect. Basic emotions, including joy, sadness, anger, fear, and disgust, that correspond to basic emotions identified by Ekman [62] were selected from Weibo-5BML to measure emotional states. The emotion of surprise was excluded because it can be both positive and negative, and it is ambiguous without context [7]. For each user given a posting, we first defined the number of positive emotion–related words (E_u_^+^) as the difference between the number of 1 positive emotion (joy) related–word and the number of 4 negative emotion (sadness, anger, fear, and disgust)–related words in equation (1). Then, we calculated the positive emotion score using the method described in steps 2 to 5. Similarly, we also estimated the number of positive sentiment–related words (S_u_^+^) by subtracting the number of positive affect–related words from the number of negative affect–related words in equation (2) and the positive sentiment score [7,50-52]. The positive sentiment score and positive emotion score are the difference between the number of positive sentiments and positive emotions and the number of negative sentiments and negative emotions in each sentence. They indicate that the overall sentiment and emotion in a given posting is rather positive or negative [50].

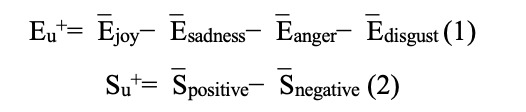



##### Step 2

The probability of depression and anxiety is higher for MSM than the general population. Therefore, understanding the frequency and distribution of MSM’s emotion-related words across hours gives the clues to find the riskiest period of their status on depression and anxiety, which provides the opportunity for various time-based interventions on mental health targeting MSM. Therefore, sentiment and emotions were measured and displayed across hours, which is similar to one study [40]*.* For each user in a given hour, we first counted the number of words of postings and the number of words related to positive affect, negative affect, positive sentiment score, joy, sadness, anger, fear, disgust, and positive emotion score in postings. Then, we calculated the following probability (eg, for calculating positive affect):

PA(u, h)=||PAWORDS(u, h)||/||WORDS(u, h)|| (3)

We used U to index the set of users (u∈U) and H to index the set of hours a day (h∈H and H={0,1,2,3…23} (assuming 0–23 for a day)). The measurement of other sentiments or emotions was computed similarly.

##### Step 3

Regarding variables to assess a user’s sentimental and emotional level, for each user, we calculated his baseline probability of affect (averaging P(u,h) across all hours):

PA=Σ_hH_ PA(u, h)/||H|| (4)

Note that the baseline probability of affect did not vary from hour to hour and therefore was an indication of the user’s average affective state. Consequently, the baseline probability of affect was used as a dependent variable in the multiple linear regression model.

##### Step 4

To measure diurnal mood rhythms, we then calculated the user’s relative probability of affect as defined in equation (5), where the last term is the grand mean across all users over all hours. The relative probability of affect represents the user’s deviation from his own baseline probability, which allows us to focus on the user’s diurnal mood rhythms by the hour of the day.

RPA(u, h)=PA(u, h)−PA+Σ_(u, h)U,H_ PA(u, h)/||UH|| (5)

##### Step 5

Finally, we calculated the general level of the relative probability affect score as defined in equation (6), where U(h) is the subset of users who were active during hour h [40].

RPA(h)=Σ_uU(h)_ RPA(u, h)/||U(h)|| (6)

#### Sexual Behaviors and Health Status

Sexual behavior–related words (eg, sex, condom, and kiss) and health-related words (eg, infection, insomnia, and exercise) from SC-LIWC were applied to identify the words associated with sexual behaviors and health status in the postings (see Table 1) [58]. The measurement of these 2 dimensions was the same as the measurement of sentiment and emotions. Baseline probabilities of sexual behaviors and health status were calculated as independent variables.

#### Themes of Postings

Understanding negative affect among MSM is critical for a mental health intervention or crisis interventions. Negative affect peaked between 2 AM and 5 AM, and therefore, 30 postings were selected randomly per time point per day to find out why users had high levels of negative affect. Finally, 840 postings were collected. To identify the themes of postings, especially the negative postings, we scanned all postings to determine the major themes [63]. These postings were categorized into the following 4 themes: (1) expression of emotion and possible cause; (2) expression of health status; (3) expression of sexual behaviors; and (4) expression of emotion. Besides, there was an additional theme categorized for the negative postings: (5) expression of emotion and seeking support.

### Statistical Analysis

First, mean and standard deviations were used to describe continuous variables. The frequency was used to describe categorical variables. Next, sentiment and emotion scores displayed a skewed distribution and, therefore, a multiple linear regression model using permutation tests that do not assume normally distributed errors was performed [64]. Univariate and multivariate linear regression models were performed to examine associations between 2 predicted variables—sexual behaviors and health status—and all outcome variables—sentiment (positive affect, negative affect, and positive sentiment score) and emotions (joy, sadness, anger, fear, disgust, and positive emotion score). Variables significant at the *P*<.20 level in the univariate analysis were included and adjusted in the multivariate linear regression model [65]. In total, 840 postings were iteratively coded and sorted into themes by 2 trained assistants separately. To ensure reliability, interrater reliability was measured, and an additional assistant was invited to code the postings that are classed as different themes. The R 3.4.3 version was applied for data analysis [66]. Statistical significance was set at *P*<.05.

## Results

### Demographic Characteristics

A total of 5871 active MSM users on Blued from the Guangdong province and their 477,374 postings were finally selected. Half of the MSM (3171/5871, 53.99%) were aged 25 years or less, and 35.29% (2072/5871) had an education beyond high school and 13.10% (769/5871) of the MSM had a high school education or below. Most MSM lived in Guangzhou (1999/5871, 34.05%), Shenzhen (1861/5871, 31.70%), or Dongguan (510/5871, 8.69%). Nearly, half of the MSM (2841/5871, 48.39%) identified their hometown as Guangdong, while 36.48% (2142/5871) of the MSM identified their hometown as non-Guangdong. Physically, 75.76% (4448/5871) of the MSM were of normal weight, 16.39% (962/5871) were underweight, 6.64% (390/5871) were overweight, and 1.21% (71/5871) were obese. Sexually, 28.92% (1698/5871) of the MSM self-reported a preference for the insertive sex role, 20.35% (1195/5871) self-reported a preference for the receptive sex role, and 22.55% (1324/5871) self-reported a preference for the versatile sex role (see Table 2).

### Affective States: Sentiment

The mean (SD) scores of positive affect, negative affect, and positive sentiment score were 0.014 (0.010), 0.016 (0.012), and −0.001 (0.015), respectively (see Table 3).

**Table 2:** Sample demographics among men who have sex with men (N=5871).

Characteristics		Value, n (%)
**Age (years)**		
	≤25	3171 (53.99)
	>25	2700 (45.99)
**Education level**		
	High school or below	769 (13.10)
	Above high school	2074 (35.33)
	Missing	3028 (51.58)
**Geolocation**		
	Guangzhou	1999 (34.05)
	Shenzhen	1861 (31.70)
	Dongguan	510 (8.69)
	Other cities in Guangdong	1501 (25.57)
**Hometown**		
	Guangdong	2841 (48.39)
	Non-Guangdong	2142 (36.48)
	Missing	888 (15.13)
**BMI classification**		
	Underweight	962 (16.39)
	Normal	4448 (75.76)
	Overweight	390 (6.64)
	Fat	71 (1.21)
**Sex role**		
	Receptive	1195 (20.35)
	Insertive	1698 (28.92)
	Versatile	1324 (22.55)
	Missing	1654 (28.17)

**Table 3:** Sentiment, emotions, social network variables, sexual behaviors, and health status among men who have sex with men (N=5871).

Characteristics		Value, mean (SD)
**Sentiment**		
	Positive affect	0.014 (0.010)
	Negative affect	0.016 (0.012)
	Positive sentiment score	−0.001 (0.015)
**Emotions**		
	Joy	0.015 (0.012)
	Sadness	0.005 (0.005)
	Anger	0.0005 (0.001)
	Fear	0.0006 (0.001)
	Disgust	0.004 (0.007)
	Positive emotion score	0.005 (0.015)
**Sexual behavior and health**		
	Sexual behaviors	0.009 (0.008)
	Health status	0.008 (0.008)
**Social network variables**		
	The number of followers (log)	2.341 (0.430)
	The number of followees (log)	1.786 (0.889)
	The number of chat groups (log)	0.277 (0.331)

#### Sentiment Diurnal Variation

**Figure 2:**
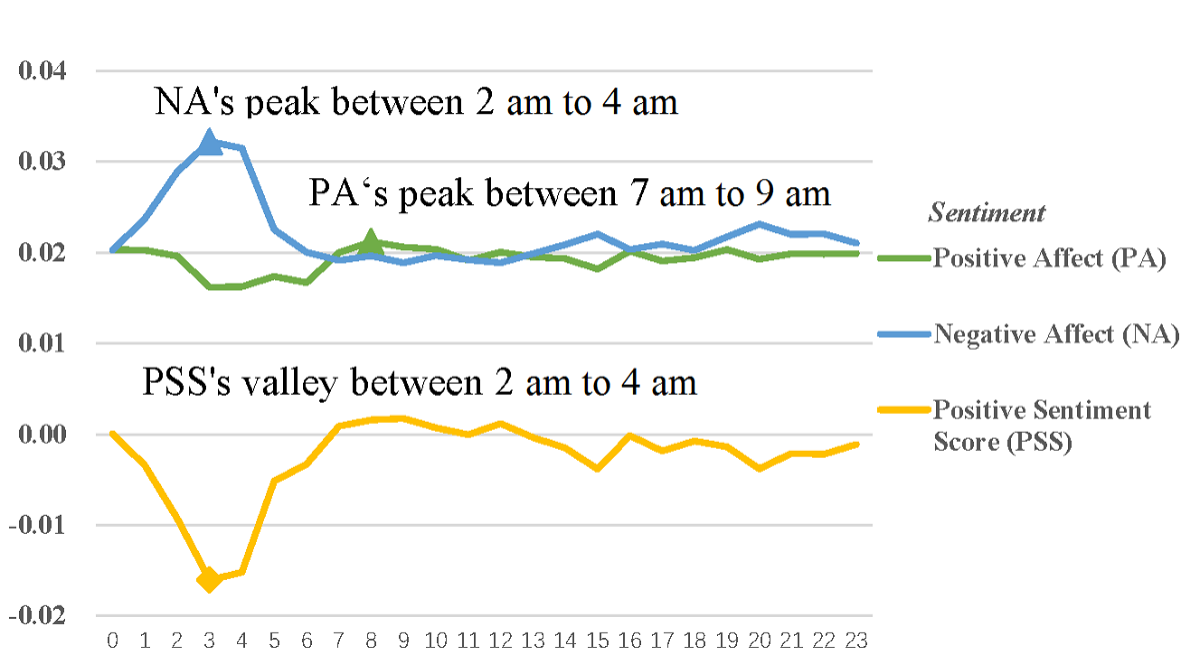
Hourly changes in individual sentiment.

Positive affect peaked between 7 AM and 9 AM, while negative affect peaked between 2 AM and 4 AM. Specifically, positive affect decreased at noon and increased at 3 AM. Then, positive affect reached the maximum between 7 AM and 9 AM, and next, positive affect showed a relatively stable trend from 9 AM to noon. Negative affect increased at noon and peaked between 2 AM and 4 AM. Then, negative affect decreased between 4 AM and 6 AM. Finally, negative affect also showed a relatively stable trend before noon. The positive sentiment score reached the minimum between 2 AM and 4 AM (Figure 2).

### Affective States: Emotion

The mean (SD) scores of joy, sadness, anger, fear, disgust, and positive emotion score were 0.015 (0.012), 0.005 (0.005), 0.0005 (0.001), 0.0006 (0.001), 0.004 (0.007), and 0.005 (0.015), respectively.

#### Emotion Diurnal Variation

**Figure 3:**
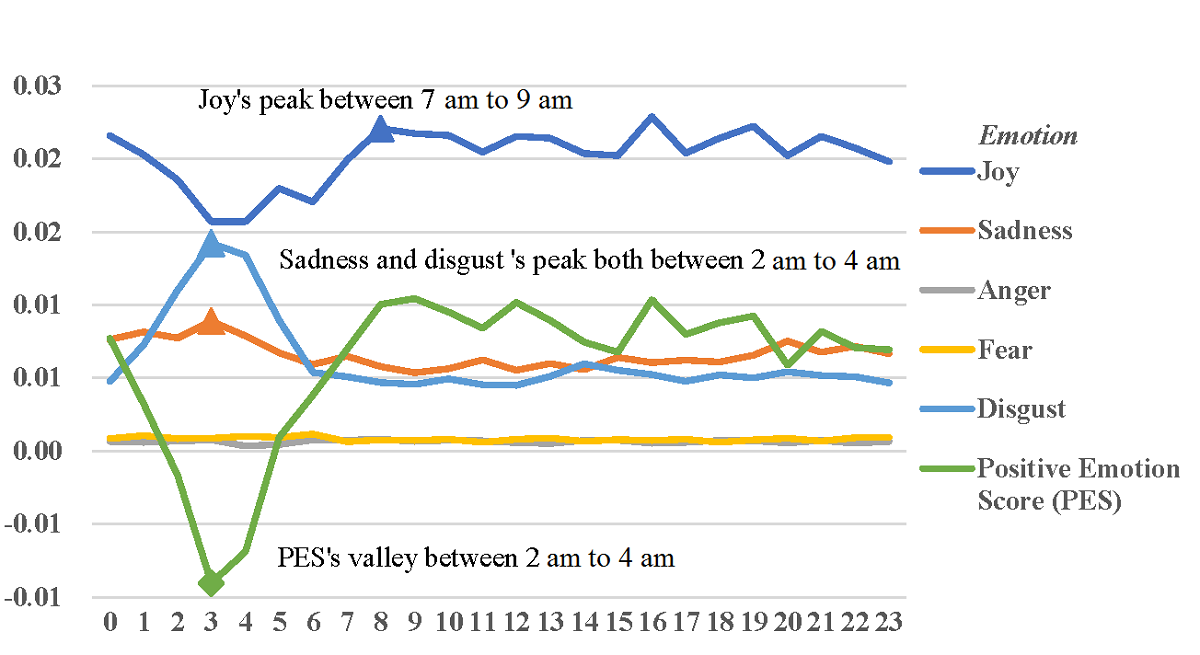
Hourly changes in individual emotions.

Joy was at a peak between 7 AM and 9 AM, while both sadness and disgust were at a peak between 2 AM and 4 AM. Specifically, joy decreased at noon and increased at 3 AM. Then, joy peaked between 7 AM and 9 AM. Next, joy showed a relatively stable trend but had 2 small peaks at 4 PM and 7 PM. Sadness and disgust both peaked between 2 AM and 4 AM, and both had a relatively stable trend before noon. Anger and fear both showed a relatively stable trend over time. The positive emotion score reached the minimum between 2 AM and 4 AM (Figure 3).

### Theme of Postings

Negative affect peaked between 2 AM and 5 AM. The proportion of negative, positive, and neutral affect was 46.1% (387/840), 33.8% (284/840), and 20.1% (169/840), respectively, during that time; 79.9% (671/840) of postings contained emotional information. Furthermore, 11.5% (97/840) of postings contained health-related information and 3.1% (26/840) of postings contained sexual behavior–related information.

Among the 387 negative postings, the kappa (agreement) values of the expression of health status, expression of emotion and seeking support, expression of sexual behaviors, expression of emotion and possible cause, and expression of emotion were 0.91 (97%), 0.63 (93%), 0.56 (98%), 0.96 (98%), and 0.92 (99%), respectively. A quarter (97/387, 25.1%) of the negative postings were directly related to health status, among which 86.6% (335/385) were on sleep health or mental health and another 13.4% (52/387) were on physical health. The expression of emotion and seeking support accounted for 13.4% (52/387), and the expression of sexual behaviors accounted for 2.6% (10/387). Of the negative postings, 55.0% (213/387) expressed strong emotion and described possible cause, among which the topics varied, from daily life events (eg, amusement and work; 109/213, 51.2%) and relationship (eg, partnership and friendship; 80/213, 37.5%) to the philosophy of life (24/213, 11.3%); 3.9% (15/387) of postings only expressed strong emotion. Example responses (see Multimedia Appendix 1B-F for the original Chinese text) included the following:

Waking up in the middle of the night suddenly and I couldn’t fall asleep at night. No one to hug me and feeling lonely.

Sleep health or mental health.

I want to find a boyfriend in Shenzhen, but ugly men couldn’t get a boyfriend.

Expression of emotion and seeking support.

Very irritated. I feel I am in a rut and I try my best to control my sexual behaviors.

Expression of sexual behaviors.

Alas, I can't seem to do anything to progress in life. Try to sleep rather than thinking which makes me upset.

Daily life event.

Even though I tried to be strong, I couldn't help but cry.

Expression of emotion.

Among the 284 positive postings, the kappa (agreement) values of the expression of health status, expression of sexual behaviors, expression of emotion and possible cause, and expression of emotion were 0.91 (99%), 0.88 (96%), 0.78 (90%), 0.79 (90%), respectively. Only 4.6% (13/284) of the positive postings were related to health status, and a quarter (71/284, 25.0%) of the positive postings were directly related to sexual behaviors. The expression of emotion accounted for 35.9% (102/284), and the expression of emotion and possible cause accounted for 34.5% (98/284). Example responses (see Multimedia Appendix 1G-J for the original Chinese text) included the following:

Gathering sand into a tower~ working, exercising, and doing charity (donation steps) ~ you can also do it.

Expression of health status; “Gathering sand into a tower” is similar as “a pin a day is a groat a year” in English. For “donation steps,” if WeChat, the most popular social network app in China, tracks your steps to be more than 10,000 per day, the WeChat company, Tencent, will donate 2 Renminbi to the charity foundation.

They said that being loved is a kind of happiness. The receptive role guyin sex has a feeling like the god in the paradise! What do you think???

Expression of sexual behaviors.

Finally saw the Russia in the snow, satisfied!

Expression of emotion and possible cause.

In a very good mood.

Expression of emotion.

### Social Network

The mean (SD) scores of followers, followees, and chat groups were 2.341 (0.430), 1.786 (0.889), and 0.277 (0.331) after log transformations, respectively (see Table 3).

### Sexual Behaviors and Health Status

**Figure 4:**
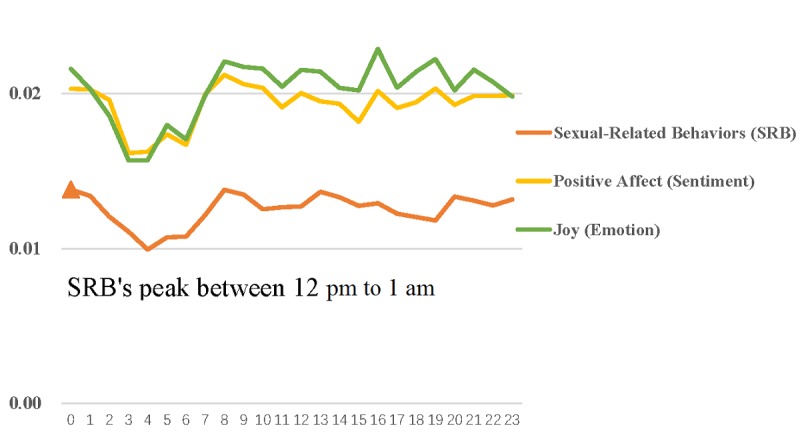
Hourly changes in individual sexual behaviors, positive affect, and joy.

**Figure 5:**
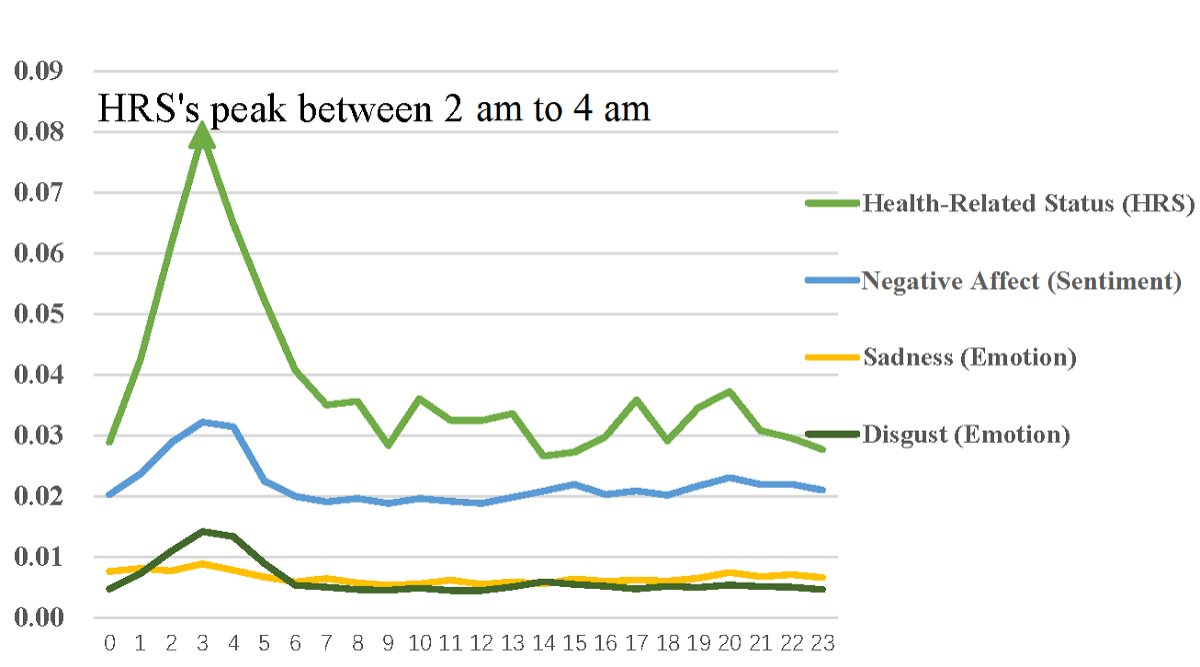
Hourly changes in individual health status, negative affect, sadness, and disgust.

The mean (SD) scores of sexual behaviors and health status were 0.009 (0.008) and 0.008 (0.008), respectively. Sexual behaviors peaked between noon and 1 AM. More importantly, there was a similar trend between sexual behaviors, positive affect, and joy (see Figure 4). Health status peaked between 2 AM and 4 AM. In addition, there was a similar trend between health status, negative affect, and disgust (see Figure 5).

### Associations Between Affective States and Demographic Characteristics

Multimedia Appendices 2-4 show the associations between affective states and demographic characteristics. Multivariable models demonstrated that compared with younger MSM (≤25 years), older MSM (>25 years) expressed more negative affect (beta=0.0004; *P*<.001), more negative emotions (anger: beta=0.00003; *P*<.001 and fear: beta=0.00003; *P*=.04), less positive affect (beta=−0.0004; *P*<.001), and a less negative emotion (disgust: beta=−0.0001; *P*=.03) and had a lower positive sentiment score (beta=−.0009; *P*<.001) and a lower positive emotion score (beta=−0.0002; *P*=.03). Compared with the MSM with a high school education or below, the MSM with an education beyond high school expressed more negative emotions (sadness: beta=0.0004; *P*=.02 and fear: beta=0.0001; *P*<.001) and had a lower positive sentiment score (beta=−0.0009; *P*=.03). Compared with the MSM who lived in Guangzhou, those who lived in Shenzhen expressed less negative emotions (sadness: beta=−0.0004; *P*<.001 and anger: beta=−0.00001; *P*=.049); the MSM who lived in Dongguan expressed less positive affect (beta=−0.0004; *P*<.001) and a less positive emotion (joy: beta=−0.0007; *P*<.001); the MSM who lived in other cities in Guangdong expressed more negative affect (beta=0.0006; *P*<.001) and a more positive emotion (joy: beta=0.001; *P*<.001). Compared with the MSM whose hometowns were in Guangdong, non-Guangdong MSM expressed a more positive emotion (joy: beta=0.0003; *P*=.02), a more negative emotion (fear: beta=0.00002; *P*=.005), less negative affect (beta=−0.0003; *P*<.001), and a less negative emotion (anger: beta=−0.00005; *P*<.001) and had a higher positive sentiment score (beta=0.0007; *P*<.001) and a higher positive emotion score (beta=0.0006; *P*=.02). Compared with the MSM with normal weight, underweight MSM expressed less positive affect (beta=−0.0004; *P*=.02) and less negative emotions (anger: beta=−0.00007; *P*<.001 and fear: beta=−0.00006; *P*=.03). Compared with the MSM with normal weight, overweight MSM expressed more negative affect (beta=0.0008; *P*<.001) and a more negative emotion (sadness: beta=0.0004; *P*<.001) and had a lower positive sentiment score (beta=−0.0008; *P*=.04). Obese MSM expressed less positive affect (beta=−0.0004; *P*=.04) and more negative affect (beta=0.0006; *P*=.03) and had a lower positive sentiment score (beta=−0.0009; *P*=.03). Compared with the MSM who prefer receptive anal intercourse, those preferring insertive anal intercourse expressed less negative affect (beta=−0.0003; *P*<.001), whereas those preferring versatile anal intercourse expressed more negative affect (beta=0.0001; *P*=.04) and a less negative emotion (fear: beta=−0.000002; *P*=.03).

### Associations Between Affective States and Social Networks

In multivariable models (Multimedia Appendices 2-4), the MSM with more chat groups expressed more positive affect (beta=0.0008; *P*<.001), a more positive emotion (joy: beta=0.0022; *P*<.001), a less negative emotion (sadness: beta=−0.0003; *P*=.03), a higher positive sentiment score (beta=0.0011; *P*<.001), and a higher positive emotion score (beta=0.0024; *P*<.001). The MSM with more followees expressed more negative emotions, including sadness (beta=0.0002; *P*<.001) and disgust (beta=0.0003; *P*=.002). The MSM with more followers expressed more positive affect (beta=0.002; *P*<.001), a more positive emotion (joy: beta=0.003; *P*<.001), a less negative emotion (sadness: beta=−0.0001; *P*=.04), a more negative emotion (disgust: beta=0.003; *P*=.02), a higher positive sentiment score (beta=0.002; *P*<.001), and a higher positive emotion score (beta=0.0026; *P*<.001).

### Associations Between Affective States and Sexual Behaviors

In multivariable models (Multimedia Appendices 2-4), the MSM who were more likely to post sexual behaviors not only expressed more positive affect (beta=0.3107; *P*<.001) and a positive emotion (joy: beta=0.027; *P*<.001) but also expressed more negative emotions, including sadness (beta=0.0443; *P*<.001) and disgust (beta=0.0256; *P*<.001). More importantly, the MSM who were more likely to post sexual behaviors had a higher positive sentiment score (beta=0.2947; *P*<.001) and a higher positive emotion score (beta=0.1612; *P*<.001).

### Associations Between Affective States and Health Status

In multivariable models (Multimedia Appendices 2-4), the MSM who were more likely to post their health status not only expressed less positive affect (beta=−0.0224; *P*=.02) but also expressed more negative affect (beta=0.8088; *P*<.001) and negative emotions, including sadness (beta=0.0705; *P*<.001), anger (beta=0.0058; *P*<.001), fear (beta=0.0052; *P*<.001), and disgust (beta=0.3065; *P*<.001). Moreover, the MSM who were more likely to post their health status had a lower positive sentiment score (beta=−0.8306; *P*<.001) and a lower positive emotion score (beta=−0.3743; *P*<.001).

## Discussion

### Principal Findings

This is one of the first studies to assess the affective states of MSM using social media data. In addition, this study is the first to explore affective states as the factor associated with sexual behaviors and health status among the MSM population.

This study showed that the MSM population was more likely to express negative affect and negative emotions (sadness and disgust) between 2 AM and 4 AM. In addition, a quarter of the negative postings were directly related to MSM’s health and about one-eighth reported that MSM needed social support during that sensitive period. Owing to the high prevalence of depression and strong links between the emotional words used and clinical depression among MSM, it is essential to implement interventions (eg, providing Web-based psychological counseling or tailored risk reduction reminders) based on this app [37-39]. Few studies have assessed the affective states of MSM based on social media, but some studies have shown that diurnal mood swings reflect endogenous circadian rhythms interacting with the duration of prior sleep or wakefulness. A previous study showed that positive affect rose quickly from 9 AM to noon and remained steady until 9 PM, after which it fell sharply [67]. In other words, positive affect peaked between midnight and 9 PM [67]. Another study found that positive emotions (happy, warm, and enjoying) had 2 peaks at noon and in the evening [68]. A time-series study found that positive affect had 2 peaks in the afternoon and evening [69]. Another study found that positive affect had 2 peaks: relatively early in the morning and again near midnight [40]. In this study, positive affect and positive emotions (eg, joy) peaked in the morning and relatively plateaued from morning to evening, which is different from other studies [40,67-70]. As for negative affect and negative emotions, studies found that negative affect peaked in the afternoon [69] and evening [40,69]. Negative emotions (eg, depressed or blue, hassled, criticized, worry, and angry) had 2 peaks at midmorning and midafternoon [68]. Besides, several studies have found that negative affect is not subject to diurnal variation [67,70]. This study found that negative affect and negative emotions (eg, sadness and disgust) peaked in the evening, which was similar to a previous study [40]. However, negative affect and negative emotions also relatively plateaued from morning to evening. There are several reasons for the diurnal variation in mood among MSM. First, for some people, the symptoms of depression may be worse at night, leading to difficulty in getting to sleep and to a feeling of isolation and hopelessness. Second, owing to prejudice, stigma, and social pressures for MSM, they may be more likely to use gay apps (eg, Blued) to express their mood in the evening. Moreover, MSM may tend to avoid discussing their health, needs, and mood in real life [71]. However, Blued provides an anonymous environment for MSM to exchange opinions and share information, which possibly explains why these men tended to talk more openly about health-related topics, express affect, and seek help [19]. These findings highlight the unmet emotional requests of MSM.

The study found that sexual behaviors were associated with positive affect, positive emotions, and negative emotions. More importantly, the MSM who were more likely to post sexual behaviors had a higher positive sentiment score and a higher positive emotion score. Previous studies found that positive affect facilitated sexual behaviors [23,72]. It is generally acknowledged that positive affect can increase sexual arousal and sexual desire and therefore facilitate sexual behavior [23]. In contrast, the MSM with negative emotions may use sex as a mood regulator and may practice more condomless anal sex with casual partners [27,73-75]. One possible explanation for this finding is that health-compromising behaviors (eg, unprotected sex) may be used as coping mechanisms to manage the effects of negative emotions. Owing to the high prevalence of HIV among MSM, practicing riskier sexual behaviors, in turn, may lead to a higher psychological burden [76]. In general, sexual behaviors seemed to be more associated with positive affect and positive emotions in this study.

Finally, the MSM who are more likely to post their health status may express more negative affect and negative emotions. Positive affect facilitates positive health behavior, leading to favorable health outcomes such as fewer symptoms and less pain [9]. Nevertheless, negative affect and negative emotions usually coexist with illness and may influence one’s functional status and health-related quality of life, meaning that measuring negative affect and negative emotions may provide a valuable means for understanding the health of MSM [77].

### Limitations

It is important to acknowledge the limitations of this study. First, we only used data from the Guangdong province, which may affect generalization owing to the cultural differences in different geographic locations. In addition, the generalizability of this study was also affected by the characteristics of the study participants. Compared with a community sample of Chinese MSM, the internet sample was significantly younger and more educated [19]. Moreover, compared with other studies using millions of users’ messages, the sample size is not big enough to measure affective states precisely [40]. Second, although keyword research was used to measure sexual behaviors and health status, social media data may not be fully representative of a user’s actual behaviors, indicating that a combination of social media data and survey research is essential to understanding the association between sexual behaviors and health status and affective states.

### Conclusions

The MSM social media community mainly expressed their positive affect in the early morning and negative affect after midnight. Positive affective states were associated with being sexually active, whereas negative affective states were associated with health problems, mostly about mental health. The findings of this study suggest different health-related intervention strategies for MSM app developers and users. MSM app developers can consider switching the banner of the app’s home page or prioritizing the posts or users over time with algorithms according to the affective states of MSM. For example, the reminders of safe sex on the home page can be displayed in the early morning, while heartwarming slogans or psychological counseling links can be exhibited in the wee hours. Furthermore, social media–based psychological assistance can narrow down to focus more on users who stably expressed negative affects, whereas the sexual risk reduction interventions can focus on those who stably expressed positive affects. For example, the content with sexual health issues could be prioritized for the users with positive sentiment states, whereas the content with encouragement could be prioritized for the users with negative sentiment states. This study shows the potential of using social media to support MSM with health issues. Future online-offline integrating surveys are warranted to confirm the outcomes of the health (eg, depression, infections, etc) and health-related behaviors (eg, sexual behaviors) of MSM for providing precision interventions to those who are most in need.

## Appendix

Multimedia Appendix 1 Quoted vocabulary and responses in the original Chinese text.

Multimedia Appendix 2 Univariate and multivariate analysis of sentiment, sexual behaviors, and health status.

Multimedia Appendix 3 Univariate and multivariate analysis of emotions (joy, sadness, and disgust), sexual behaviors, and health status.

Multimedia Appendix 4 Univariate and multivariate analysis of emotions (anger, fear, and positive emotion score), sexual behaviors, and health status.

## Abbreviations

ADB: android debug bridge
API: application programming interface
MSM: men who have sex with men
OCR: optical character recognition
SC-LIWC: Simplified Chinese Linguistic Inquiry and Word Count
Weibo-5BML: Weibo Basic Mood Lexicon
