# Correction: Associations Between Affective States and Sexual and Health Status Among Men Who Have Sex With Men in China: Exploratory Study Using Social Media Data

**DOI:** 10.2196/18135

**Published:** 2020-03-03

**Authors:** Zhi-Wei Zheng, Qing-Ling Yang, Zhong-Qi Liu, Jia-Ling Qiu, Jing Gu, Yuan-Tao Hao, Chao Song, Zhong-Wei Jia, Chun Hao

**Affiliations:** 1 Department of Medical Statistics School of Public Health Sun Yat-sen University Guangzhou China; 2 Health Information Research Center, Guangdong Key Laboratory of Medicine School of Public Health Sun Yat-sen University Guangzhou China; 3 Sun Yat-sen Global Health Institute Institute of State Governance Sun Yat-sen University Guangzhou China; 4 School of Computer Science and Engineering University of Electronic Science and Technology of China Chengdu China; 5 National Institute on Drug Dependence Peking University Beijing China

In “Associations Between Affective States and Sexual and Health Status Among Men Who Have Sex With Men in China: Exploratory Study Using Social Media Data” (J Med Internet Res 2020;22(1):e13201), there was an error which was not identified during the proofing stage.

The original published Acknowledgments section was incorrectly listed as:

We are grateful to the participants of this study. This project was funded by the National Natural Science Foundation of China (grant number #71974212, #71774178, and #81803334), Science and Technology Program of Guangzhou, China (grant number #201607010332), and A Major Infectious Disease Prevention and Control of the National Science and Technology Major Project (grant number #2018ZX10715004).

The correct Acknowledgments section is:

We are grateful to the participants of this study. This project was funded by the National Natural Science Foundation of China (grant number #91546203, #71974212, #71774178, and #81803334), Science and Technology Program of Guangzhou, China (grant number #201607010332) and A Major Infectious Disease Prevention and Control of the National Science and Technology Major Project (grant number #2018ZX10715004).

The correction will appear in the online version of the paper on the JMIR website on March 3, 2020, together with the publication of this correction notice. Because this was made after submission to PubMed, PubMed Central, and other full-text repositories, the corrected article has also been resubmitted to those repositories.

