# Supplementary material for: Associations Between Affective States and Sexual and Health Status Among Men Who Have Sex With Men in China: Exploratory Study Using Social Media Data
Source: J Med Internet Res. 2020 Jan 31;22(1):e13201. doi: 10.2196/13201 (PMC7053714; doi:10.2196/13201)
Supplement: Multimedia Appendix 1 [file jmir_v22i1e13201_app1.docx]

Multimedia Appendix 1. Quoted vocabulary and responses in the original Chinese.

**Multimedia Appendix 1A**

Table 1. Summary of the Simplified Chinese Linguistic Inquiry and Word Count and Weibo Basic Mood Lexicon dimensions used for this study and example vocabulary.

| Dimensions | | Dictionary | Number of words | Example vocabulary |
| --- | --- | --- | --- | --- |
| **Sentiment** | | | | |
|  | Positive affect | SC-LIWC^a^ | 483 | Honor (荣誉), sweet (甜蜜), happy (幸福) |
|  | Negative affect | SC-LIWC | 812 | Hurt (伤害), agony (苦恼), nasty (恶意的) |
| **Emotion** | | | | |
|  | Joy | Weibo-5BML^b^ | 306 | Love (疼爱), excite (激动), high (高昂) |
|  | Sadness | Weibo-5BML | 205 | Anxious (犯愁), alone (孤单), tear (泪水) |
|  | Anger | Weibo-5BML | 93 | Enemy (敌人), abuse (谩骂), roar (咆哮) |
|  | Fear | Weibo-5BML | 72 | Sit on pins and needles (如坐针毡), panic (惊慌), hell (地狱) |
|  | Disgust | Weibo-5BML | 142 | Wordy (啰嗦), speechless (无语), ridicule (嘲笑) |
| **Other** | | | | |
|  | Sexual-related words | SC-LIWC | 117 | Sex (性爱), condom (安全套), kiss (亲吻) |
|  | Health-related words | SC-LIWC | 375 | Infection (感染), insomnia (失眠), exercise (运动) |

^a^SC-LIWC: Simplified Chinese Linguistic Inquiry and Word Count.

^b^Weibo-5BML: Weibo Basic Mood Lexicon.

**Multimedia Appendix 1B**

*Waking up in the middle of the night suddenly and I couldn’t fall asleep at night. No one to hug me and feeling lonely* (半夜突然醒来，再也无法入眠。没有一个人的旁边抱着，感觉很孤独).

**Multimedia Appendix 1C**

*I want to find a boyfriend in Shenzhen, but ugly men couldn’t get a boyfriend* (满深圳求男友，可丑男一枚找不到男朋友).

**Multimedia Appendix 1D**

*Very irritated. I feel I am in a rut and I try my best to control my sexual behaviors* (好烦, 仿佛到了发情期, 得拼命用理智控制自己).

**Multimedia Appendix 1E**

*Alas, I can't seem to do anything to progress in life. Try to sleep rather than thinking which makes me upset* (哎, 啥求事都干不成, 睡吧, 想多了心烦).

**Multimedia Appendix 1F**

*Even though I tried to be strong, I couldn't help but cry* (心里再明白再坚强眼泪又是止不住).

**Multimedia Appendix 1G**

*Gathering sand into a tower~ working, exercising, and doing charity (donation steps) ~ you can also do it* (聚沙成塔～边工作，边锻炼，边做慈善（捐步数）～你也可以做到哦).

**Multimedia Appendix 1H**

*They said that being loved is a kind of happiness. The receptive role guyin sex has a feeling like the god in the paradise! What do you think??? (他们说，被爱是一种幸福。受在性爱上有种神仙般感觉!你说呢???)*

**Multimedia Appendix 1I**

*Finally saw the Russia in the snow, satisfied!* (终于看到了雪中的俄羅斯，满足!).

**Multimedia Appendix 1J**

*In a very good mood* (心情杠杠的好).
