# Supplementary material for: Associations Between Affective States and Sexual and Health Status Among Men Who Have Sex With Men in China: Exploratory Study Using Social Media Data
Source: J Med Internet Res. 2020 Jan 31;22(1):e13201. doi: 10.2196/13201 (PMC7053714; doi:10.2196/13201)
Supplement: Multimedia Appendix 2 [file jmir_v22i1e13201_app2.docx]

Multimedia Appendix 2. Univariate and multivariate analysis of sentiment, sexual behaviors, and health status.

| Variables or covariants | | | | Positive affect | | Negative affect | | Positive sentiment score | |
| --- | --- | --- | --- | --- | --- | --- | --- | --- | --- |
|  |  |  |  | Univariate analysis | Multivariate analysis | Univariate analysis | Multivariate analysis | Univariate analysis | Multivariate analysis |
|  |  |  |  | β | β (standardized β) | β | β (standardized β) | β | β (standardized β) |
|  |  |  |  |  |  |  |  |  |  |
| Sexual behaviors | | | | 0.3171^a^ | 0.3107 (0.266)^a^ | 0.01 | 0.0153 (0.011) | 0.3072–^a^ | 0.2947 (0.162)^a^ |
| Health status | | | | −0.0233^b^ | −0.0224 (−0.018)^b^ | 0.8079^a^ | 0.8088 (0.547)^a^ | −0.8312^a^ | −0.8306 (−0.435)^a^ |
| **Demographic characteristics** | | | | | | | | | |
|  | Age（"＞25y"） | | ref= “≤25y” | −0.0002^a^ | −0.0004 (−0.045)^a^ | 0.0006^a^ | 0.0004 (0.038)^a^ | −0.0008^a^ | −0.0009 (−0.060)^a^ |
|  | **Educational level** | | | | | | | | |
|  |  | Above high school | ref= High school or below | −0.0001 | −0.0003 (−0.028)^c^ | 0.0008^a^ | 0.0006 (0.049) | −0.0009 | −0.0009 (−0.058)b |
|  |  | Unknown | ref= High school or below | 0.0001^c^ | 0.00006 (0.006) | −0.0011^a^ | −0.0009 (−0.078)^a^ | 0.0012^a^ | 0.0010 (0.067)^a^ |
|  | **Geolocation** | | | | | | | | |
|  |  | Shenzhen | ref= Guangzhou | 0.0002 | 0.00006 (0.006) | −0.0004 | −0.0004 (−0.031) | 0.0006 | —^e^ |
|  |  | Dongguan | ref= Guangzhou | −0.0002^c^ | −0.0004 (−0.038)^a^ | −0.0003 | −0.0002 (−0.014) | 0.00007 | —^e^ |
|  |  | Other cities in Guangdong | ref= Guangzhou | 0.0001 | 0.00008 (0.009) | 0.0005^c^ | 0.0006 (0.052)^a^ | −0.0004 | —^e^ |
|  | **Hometown** | | | | | | | | |
|  |  | Non-Guangdong | ref=Guangdong | 0.0004^a^ | 0.0004 (0.035)^c^ | −0.0003^b^ | −0.0003 (−0.026)^a^ | 0.0006^a^ | 0.0007 (0.046)^a^ |
|  |  | Unknown | ref=Guangdong | 0.0001 | −0.00007 (−0.007) | −0.0001 | 0.0002 (0.015)^b^ | 0.0002 | −0.0004 (−0.023^b^ |
|  | **BMI classification** | | | | | | | | |
|  |  | Underweight | ref=Normal weight | −0.0005^b^ | −0.0004 (−0.041)^b^ | −0.0005^a^ | 0.0002 (0.014)^c^ | −0.000001 | −0.0006 (−0.038)^c^ |
|  |  | Overweight | ref=Normal weight | −0.0001 | 0.00008 (0.008) | 0.0005^c^ | 0.0008 (0.069)^a^ | −0.0006^b^ | −0.0008 (−0.053)^b^ |
|  |  | Obese | ref=Normal weight | −0.0003^b^ | −0.0004 (−0.038)^b^ | 0.0002^c^ | 0.0006 (0.050)^b^ | −0.0005^c^ | −0.0009 (−0.062)^b^ |
|  | **Sex role** | | | | | | | | |
|  |  | Versatile | ref=Receptive | 0.0002 | —^e^ | 0.0002^d^ | 0.0001 (0.009)^b^ | 0.00004 | —^e^ |
|  |  | Insertive | ref=Receptive | −0.0004 | —^e^ | −0.0004^a^ | −0.0003 (−0.029)^a^ | 0.00001 | —^e^ |
|  |  | Unknown | ref=Receptive | −0.0001 | —^e^ | −0.0005 | −0.0005 (−0.041)^a^ | 0.0004 | —^e^ |
|  | **Social network variables** | | | | | | | | |
|  |  | Number of chat groups (Log) | | 0.0014^a^ | 0.0008 (0.026)^a^ | −0.0003 | —^e^ | 0.0017^a^ | 0.0011 (0.023)^a^ |
|  |  | Number of followees (Log) | | 0.0003^a^ | 0.000004 (0.0003) | 0.0002^b^ | 0.00007 (0.005)^c^ | 0.0001 | —^e^ |
|  |  | Number of followers (Log) | | 0.0025^a^ | 0.0020 (0.087)^a^ | 0.00004 | —^e^ | 0.0024^a^ | 0.0020 (0.057)^a^ |
| Model fitting | | | | —^e^ | F=34.4, *P< .001*, adjR^2^=0.08 | —^e^ | F=153, *P< .001*, adjR^2^=0.31 | —^e^ | F=144.5, *P< .001*, adjR^2^=0.23 |

^a^*P*<.001.

^b^*P*<.05.

^c^*P*<.02.

^d^*P*<.01.

^e^ not applicable
