# Supplementary material for: Associations Between Affective States and Sexual and Health Status Among Men Who Have Sex With Men in China: Exploratory Study Using Social Media Data
Source: J Med Internet Res. 2020 Jan 31;22(1):e13201. doi: 10.2196/13201 (PMC7053714; doi:10.2196/13201)
Supplement: Multimedia Appendix 3 [file jmir_v22i1e13201_app3.docx]

Multimedia Appendix 3. Univariate and multivariate analysis of emotions (joy, sadness, and disgust), sexual behaviors, and health status.

| Variables or covariants | | | | Joy | | Sadness | | Disgust | |
| --- | --- | --- | --- | --- | --- | --- | --- | --- | --- |
|  |  |  |  | Univariate analysis | Multivariate analysis | Univariate analysis | Multivariate analysis | Univariate analysis | Multivariate analysis |
|  |  |  |  | β | β (standardized β) | β | β (standardized β) | β | β (standardized β) |
|  |  | | |  |  |  |  |  |  |
| Sexual behaviors | | | | 0.2440^a^ | 0.0270 (0.154)^a^ | 0.0451^a^ | 0.0443 (0.069)^a^ | 0.0214^a^ | 0.0256 (0.030)^a^ |
| Health related status | | | | 0.0164 | 0.0156 (0.010) | 0.0697^a^ | 0.0705 (0.105)^a^ | 0.3078^a^ | 0.3065 (0.339)^a^ |
| **Demographic characteristics** | | | |  |  |  |  |  |  |
|  | Age（"＞25y"） | | ref= “≤25y” | −0.0001 | —^e^ | 0.0002^a^ | 0.00004 (0.008)^b^ | −0.0002^c^ | −0.0001 (−0.016)^c^ |
|  | **Educational level** | | | | | | | | |
|  |  | Above high school | ref= High school or below | 0.0002 | —^e^ | 0.0005 | 0.0004 (0.066)^c^ | 0.00007^a^ | 0.00009 (0.013) |
|  |  | Unknown | ref= High school or below | −0.00003 | —^e^ | −0.0004^a^ | −0.0004 (−0.064)^a^ | −0.0004 | −0.0005 (−0.066)^a^ |
|  | **Geolocation** | | | | | | | | |
|  |  | Shenzhen | ref= Guangzhou | −0.00003^b^ | −0.0002 (−0.015) | −0.0005^a^ | −0.0004 (−0.083)^a^ | −0.0002 | −0.0001 (−0.019) |
|  |  | Dongguan | ref= Guangzhou | −0.0004 | −0.0007 (−0.058)^a^ | −0.0001 | −0.00009 (−0.017)^b^ | 0.00003 | 0.00004 (0.006) |
|  |  | Other cities in Guangdong | ref= Guangzhou | 0.0009^b^ | 0.0010 (0.085)^a^ | 0.0004^a^ | 0.0003 (0.052) | 0.0002^c^ | 0.0002 (0.032) |
|  | **Hometown** | | | | | | | | |
|  |  | Non-Guangdong | ref=Guangdong | 0.0003^a^ | 0.0003 (0.022)^c^ | −0.00007 | −0.0001 (−0.019)^b^ | −0.0002^c^ | −0.0001 (−0.015)^b^ |
|  |  | Unknown | ref=Guangdong | 0.0005^a^ | 0.0002 (0.017)^b^ | 0.0002^a^ | 0.0002 (0.042)^a^ | 0.00005 | 0.0002 (0.021) |
|  | **BMI classification** | | | | | | | | |
|  |  | Underweight | ref=Normal weight | −0.0002 | −0.0001 (−0.011) | −0.0001 | −0.00003 (−0.005) | −0.0004^c^ | −0.0002 (−0.030) |
|  |  | Overweight | ref=Normal weight | −0.0001 | −0.0001 (0.009) | 0.0005^a^ | 0.0004 (0.076)^a^ | −0.0006^a^ | −0.0002 (−0.035) |
|  |  | Obese | ref=Normal weight | −0.0005^#^ | −0.0003 (−0.025) | −0.0002 | −0.0002 (−0.038) | 0.00002 | 0.00004 (0.005) |
|  | **Sex role** | | | | | | | | |
|  |  | Versatile | ref=Receptive | −0.00007^b^ | 0.0001 (0.005) | 0.0003 | 0.0003 (0.051) | −0.0001^c^ | 0.00002 (0.003) |
|  |  | Insertive | ref=Receptive | −0.0005 | −0.0003 (−0.025) | −0.00006^d^ | −0.00008 (−0.014) | −0.00005 | 0.000 (0.000) |
|  |  | Unknown | ref=Receptive | 0.0002^c^ | −0.0001 (−0.010)^c^ | −0.0004^a^ | −0.0003 (−0.052)^b^ | 0.000007 | −0.0002 (−0.021) |
|  | **Social network variables** | | | | | | | | |
|  |  | Number of chat groups (Log) | | 0.0029^a^ | 0.0022 (0.058)^a^ | −0.0002^c^ | −0.0003 (−0.017)^c^ | 0.0001 | —^e^ |
|  |  | Number of followees (Log) | | 0.0004^a^ | −0.0001 (−0.005) | 0.0002^a^ | 0.0002 (0.036)^a^ | 0.0004^a^ | 0.0003 (0.039)^d^ |
|  |  | Number of followers (Log) | | 0.0036^a^ | 0.0030 (0.105)^a^ | −0.0001^b^ | −0.0001 (−0.010)^c^ | 0.0005^a^ | 0.0003 (0.015)^c^ |
| Model fitting | | | | —^e^ | F=17.5, *P< .001*, adjR^2^=0.04 | —^e^ | F=8.46, *P< .001*, adjR^2^=0.02 | —^e^ | F=45.69, *P< .001*, adjR^2^=0.12 |

^a^*P*<.001.

^b^*P*<.02.

^c^*P*<.05.

^d^*P*<.01.

^e^ not applicable
