# Supplementary material for: Associations Between Affective States and Sexual and Health Status Among Men Who Have Sex With Men in China: Exploratory Study Using Social Media Data
Source: J Med Internet Res. 2020 Jan 31;22(1):e13201. doi: 10.2196/13201 (PMC7053714; doi:10.2196/13201)
Supplement: Multimedia Appendix 4 [file jmir_v22i1e13201_app4.docx]

Multimedia Appendix 4. Univariate and multivariate analysis of emotions (anger, fear, and positive emotion score), sexual behaviors, and health status.

| Variables or covariants | | | | Anger | | Fear | | Positive emotions score | |
| --- | --- | --- | --- | --- | --- | --- | --- | --- | --- |
|  |  |  |  | Univariate analysis | Multivariate analysis | Univariate analysis | Multivariate analysis | Univariate analysis | Multivariate analysis |
|  | | | | β | β (standardized β) | β | β (standardized β) | β | β (standardized β) |
|  | | | |  |  |  |  |  |  |
| Sexual behaviors | | | | −0.00009 | 0.00003 (0.0002) | −0.0009 | −0.0014 (−0.008) | 0.1784^a^ | 0.1612 (0.089)^a^ |
| Health related status | | | | 0.0060^a^ | 0.0058 (0.038)^a^ | 0.0054^a^ | 0.0052 (0.029)^a^ | −0.3726^a^ | −0.3743 (−0.197)^a^ |
| **Demographic characteristics** | | | | | | | | | |
|  | Age ("＞25y") | | ref= “≤25y” | 0.00002^b^ | 0.00003 (0.024)^a^ | 0.00004^a^ | 0.00003 (0.023)^c^ | −0.0002 | −0.0002 (−0.016)^c^ |
|  | **Educational level** | | | | | | | | |
|  |  | Above high school | ref= High school or below | −0.000001^c^ | 0.000 (−0.002) | 0.00008^b^ | 0.0001 (0.043)^a^ | −0.0005^d^ | −0.0005 (−0.033) |
|  |  | Unknown | ref= High school or below | −0.00002^b^ | −0.00002 (−0.015)^c^ | −0.00006^a^ | −0.00004 (−0.030) | 0.0009^a^ | 0.0007 (0.044)^d^ |
|  | **Geolocation** | | | | | | | | |
|  |  | Shenzhen | ref= Guangzhou | −0.00001^b^ | −0.00001 (−0.005)^c^ | −0.00006^a^ | −0.00006 (−0.039) | 0.0007^a^ | 0.0005 (0.034) |
|  |  | Dongguan | ref= Guangzhou | 0.0000006 | −0.00001 (−0.006)^b^ | −0.00002^b^ | −0.00001 (−0.006) | −0.0003 | −0.0006 (−0.041) |
|  |  | Other cities in Guangdong | ref= Guangzhou | −0.00002^b^ | −0.00003 (−0.023)^b^ | 0.00001 | 0.00001 (0.008) | 0.0004 | 0.0004 (0.030) |
|  | **Hometown** | | | | | | | | |
|  |  | Non-Guangdong | ref=Guangdong | −0.00005^a^ | −0.00005 (−0.042)^a^ | 0.00003^a^ | 0.00002 (0.016)^d^ | 0.0006^a^ | 0.0006 (0.037)^c^ |
|  |  | Unknown | ref=Guangdong | 0.00003^c^ | 0.00005 (0.039)^a^ | −0.00001 | 0.000 (−0.002) | 0.0003 | −0.0002 (−0.011) |
|  | **BMI classification** | | | | | | | | |
|  |  | Underweight | ref=Normal weight | −0.00006^c^ | −0.00007 (−0.056)^a^ | −0.00007^c^ | −0.00006 (−0.045)^c^ | 0.0004 | —^e^ |
|  |  | Overweight | ref=Normal weight | −0.00007^b^ | −0.00007 (−0.054)^b^ | −0.00003 | −0.00006 (−0.042) | 0.0002 | —^e^ |
|  |  | Obese | ref=Normal weight | −0.00004^b^ | −0.00003 (−0.029) | −0.00007 | −0.00005 (−0.035) | −0.0001 | —^e^ |
|  | **Sex role** | | | | | | | | |
|  |  | Versatile | ref=Receptive | −0.00004 | −0.00004 (−0.035) | 0.00001 | −0.000002 (−0.002)^c^ | −0.0002 | −0.0001 (−0.007) |
|  |  | Insertive | ref=Receptive | −0.00002 | −0.00001 (−0.012) | −0.00001^b^ | −0.00001 (−0.009)^b^ | −0.0004 | −0.0003 (−0.018) |
|  |  | Unknown | ref=Receptive | 0.00007^a^ | 0.00009 (0.072)^a^ | −0.00005^b^ | −0.00004 (−0.026) | 0.0006^c^ | −0.0002 (0.014) |
|  | **Social network variables** | | | | | | | | |
|  |  | Number of chat groups (Log) | | −0.00009^b^ | −0.00009 (−0.023)^b^ | 0.00003 | —^e^ | 0.0031^a^ | 0.0024 (0.052)^a^ |
|  |  | Number of followees (Log) | | −0.00001 | —^e^ | −0.00002 | —^e^ | −0.0002 | —^e^ |
|  |  | Number of followers (Log) | | 0.00005 | —^e^ | 0.00001 | —^e^ | 0.0031^a^ | 0.0026 (0.073)^a^ |
| Model fitting | | | | —^e^ | F=2.1, *P*<.01, adjR^2^=0.003 | —^e^ | F=1.8, *P*=.05, adjR^2^=0.002 | —^e^ | F=25.3, *P< .001*, adjR^2^=0.06 |

^a^*P*<.001.

^b^*P*<.02.

^c^*P*<.05.

^d^*P*<.01

^e^ not applicable
